# Supplementary material for: A cancer rainbow mouse for visualizing the functional genomics of oncogenic clonal expansion
Source: Nat Commun. 2019 Dec 2;10:5490. doi: 10.1038/s41467-019-13330-y (PMC6889384; doi:10.1038/s41467-019-13330-y)
Supplement: Supplementary file 2 — Supplementary Information [file 41467_2019_13330_MOESM2_ESM.pdf]

A Cancer rainbow mouse for visualizing the functional genomics of oncogenic clonal expansion

Boone, et.al.

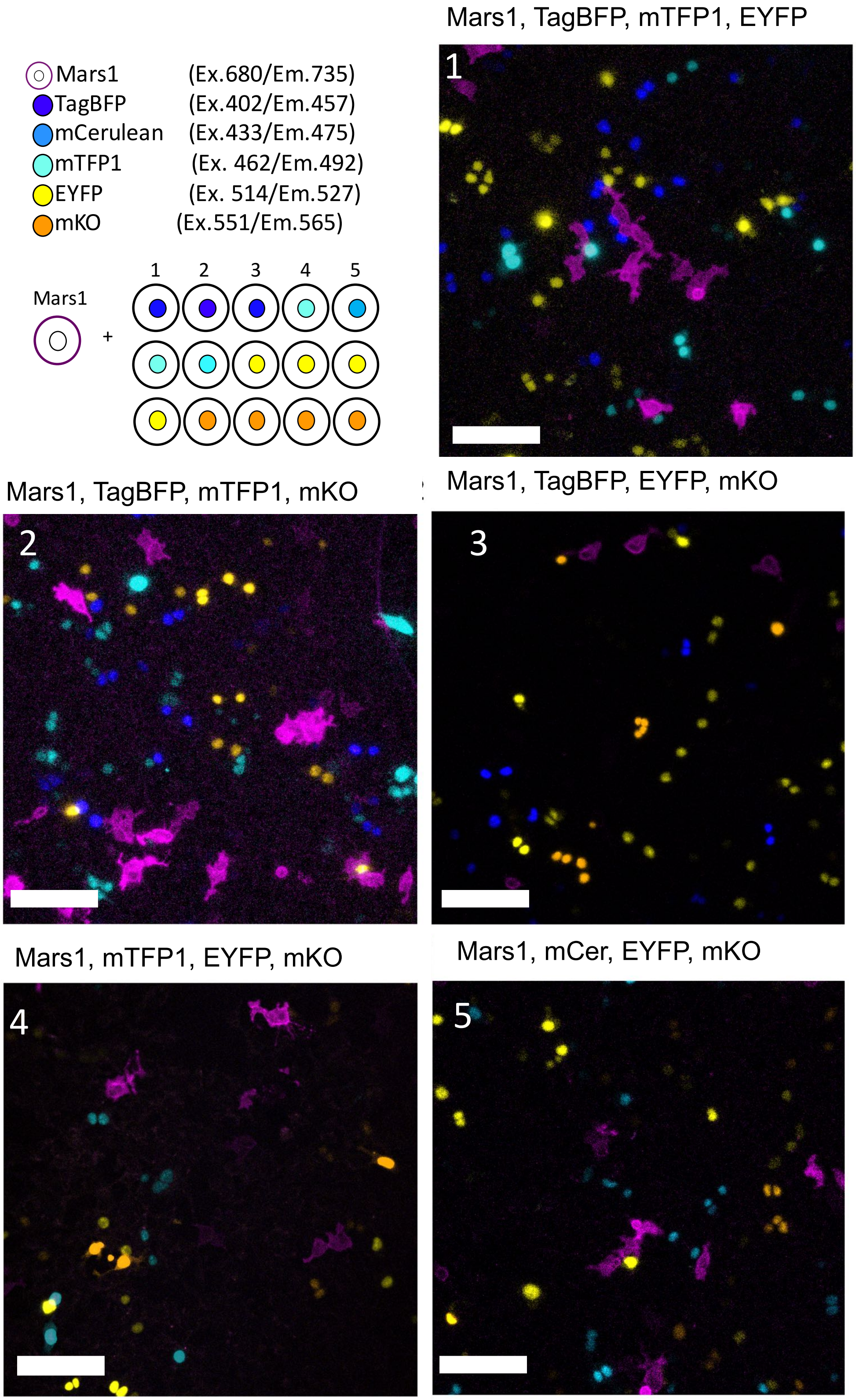

Supplementary Figure 1. Testing XFP palettes *in vitro*. The indicated nuclear fluorescent proteins and membrane targeted Mars1 were cloned into eukaryotic expression vectors and transfected alone into HEK cells. HEK cells were then mixed one-to-one in five different combinations, stained with SCi1, fixed, and imaged by confocal microscopy. Scale bars = 10μm.

Pos0: Mars1-pENTR

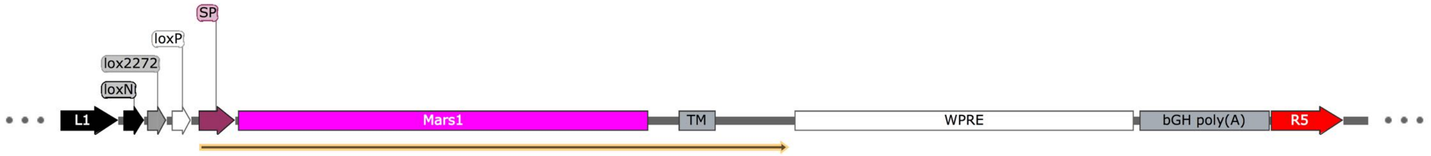

Pos1: 3X-NLS-TagBFP-2a-Infusion pENTR

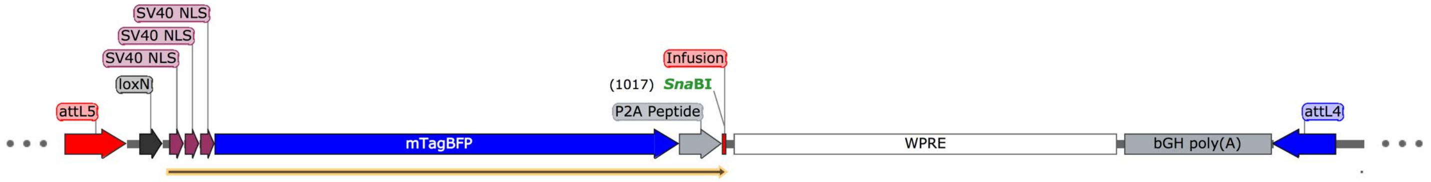

Pos2:3X-NLS-mTFP1-2a-Infusion pENTR

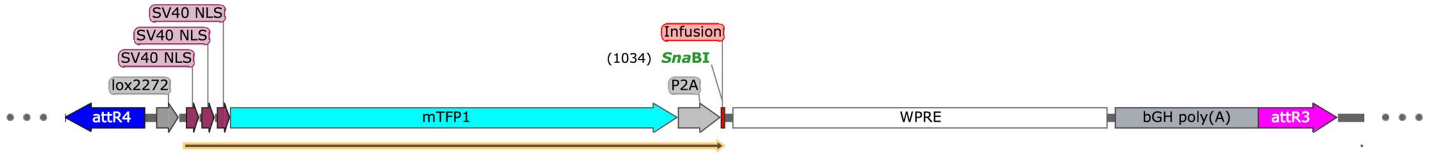

Pos3:3X-NLS-mKO-2a-Infusion pENTR

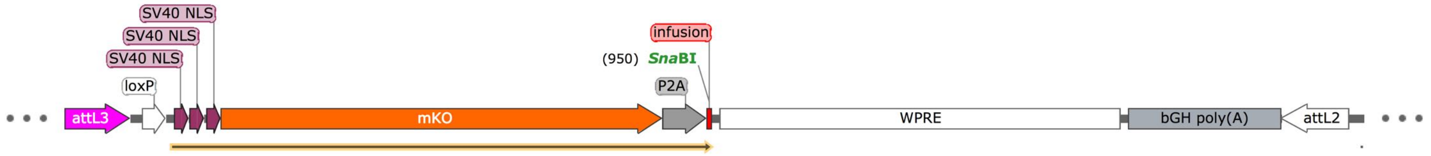

Supplementary Figure 2. pENTR vector kit for building Crainbow FBTK (**F**AP-MARS1, mTag**B**FP, m**T**FP1, m**K**O) mice. pENTR vectors compatible with four fragment Multi-site Gateway™ cloning were adapted for plug-and-play assembly of Crainbow vectors. All pENTR vectors are flanked by appropriate att-recombination sites and include a WPRE for enhancing expression. Position 0 includes loxN (black), lox2272 (grey), and loxP (white) sites and encodes a transmembrane FAP-Mars1 (magenta). Positions 1-3 encode mTagBFP (blue), mTFP1 (cyan), and mKO (orange), and include loxN, lox2272, and loxP, respectively. Additionally, positions 1-3 include a single SnaBI restriction site for linearization and in-frame Infusion® cloning of driver genes produced by gene synthesis or PCR amplification. pENTR vectors with the appropriate driver gene were ready for single-step and directional cloning into a Gateway™-compatible ROSA-targeting vector (**Supplementary Figure 4**).

Pos1: Mars1-pENTR

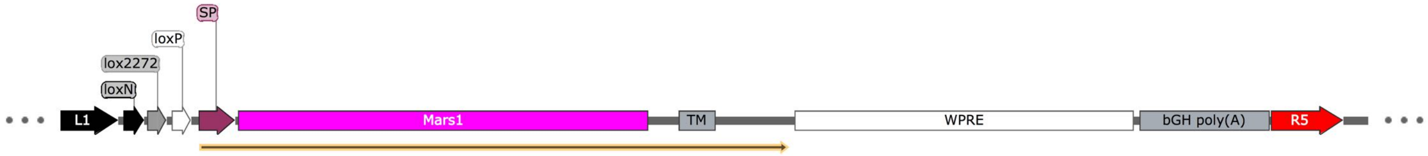

Pos2: 3X-NLS TagBFP-2a-Infusion pENTR

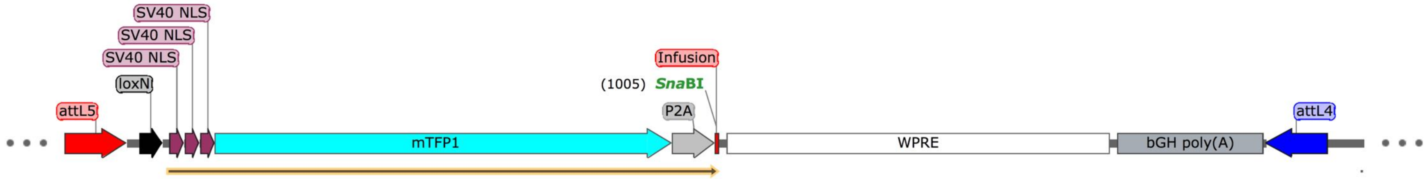

Pos3:3X-NLS-EYFP-2a-Infusion pENTR

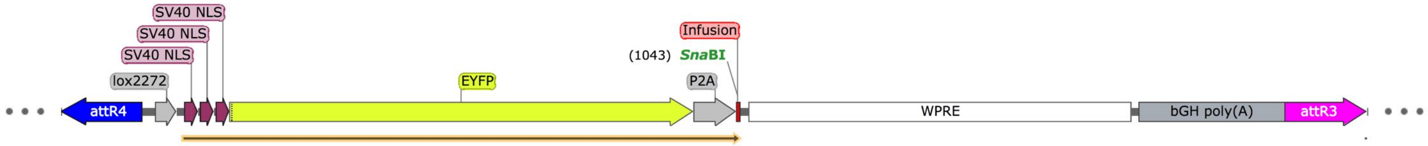

Pos4:3X-NLS-mKO-2a-Infusion pENTR

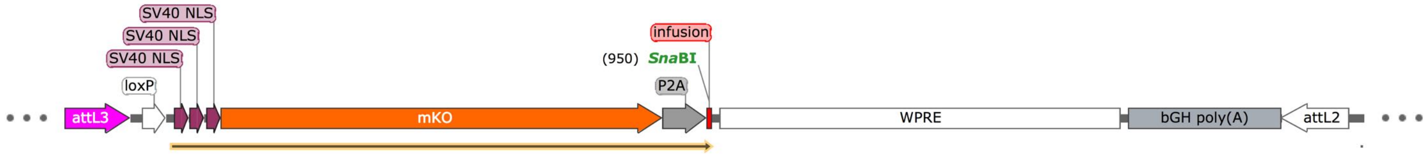

Supplementary Figure 3. pENTR vector kit for building Crainbow F̲TYK (**F̲**AP-MA̲RS1,m**T**FP1, E**Y**FP m**K**O) mice. pENTR vectors compatible with four-fragment Multi-site Gateway™ cloning were adapted for plug-and-play assembly of Crainbow vectors. All pENTR vectors are flanked by appropriate att-recombination sites and include a WPRE for enhancing expression. Position 0 includes loxN (black), lox2272 (grey), and loxP (white) sites, and encodes a transmembrane FAP-Mars1 (magenta). Positions 1-3 encode mTFP1 (cyan), EYFP (yellow), and mKO
(orange), and include loxN, lox2272, and loxP, respectively. Additionally, positions 1-3 include a single SnaBI restriction site for linearization and in-frame Infusion® cloning of driver genes produced by gene synthesis or PCR amplification. pENTR vectors with the appropriate driver gene were ready for single-step and directional cloning into a Gateway™-compatible ROSA-targeting vector (**Supplementary Figures 5 and 7**).

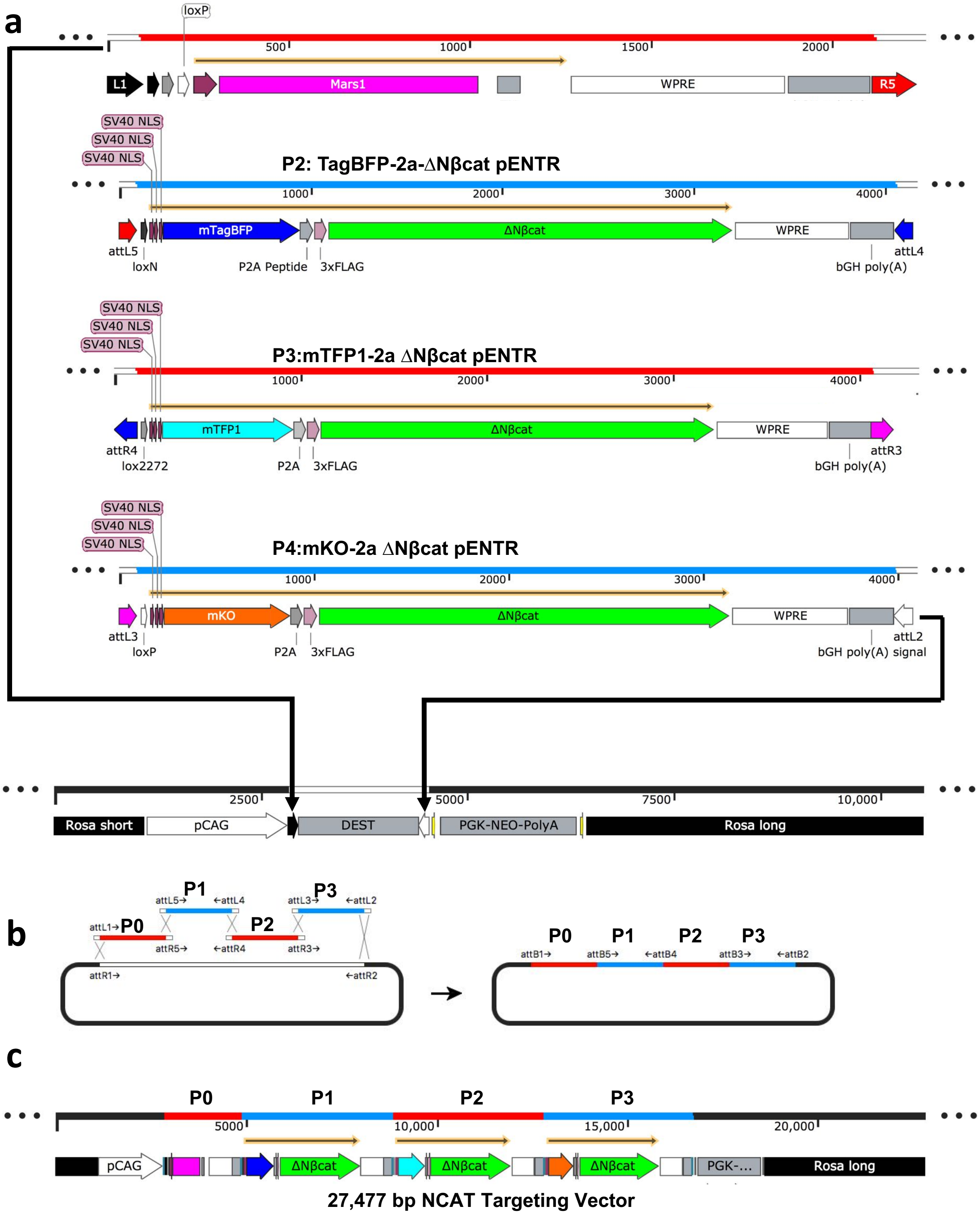

Supplementary Figure 4. Genetic map of NCAT Crainbow mice. (a) FBTK pENTR vectors were used to assemble a ROSA-targeting vector for a ΔNßcat control Crainbow mouse model (NCAT). cDNA encoding 3x-FLAG-tagged and N-terminally truncated mouse ßcatenin (Uniprot Q02248, aa79-781) was Infusion® cloned into P2, P3, and P4 FBTK pENTR vectors. **(b)** Single-step multi-fragment Gateway™ assembly of FBTK pENTR vectors from **"a"** into a previously described Gateway™-compatible ROSA-targeting mouse vector[1]. **(c)** ROSA NCAT Crainbow vectors were restriction mapped, sequence verified using next-generation sequencing (NGS), and linearized for ES cell electroporation and selection.

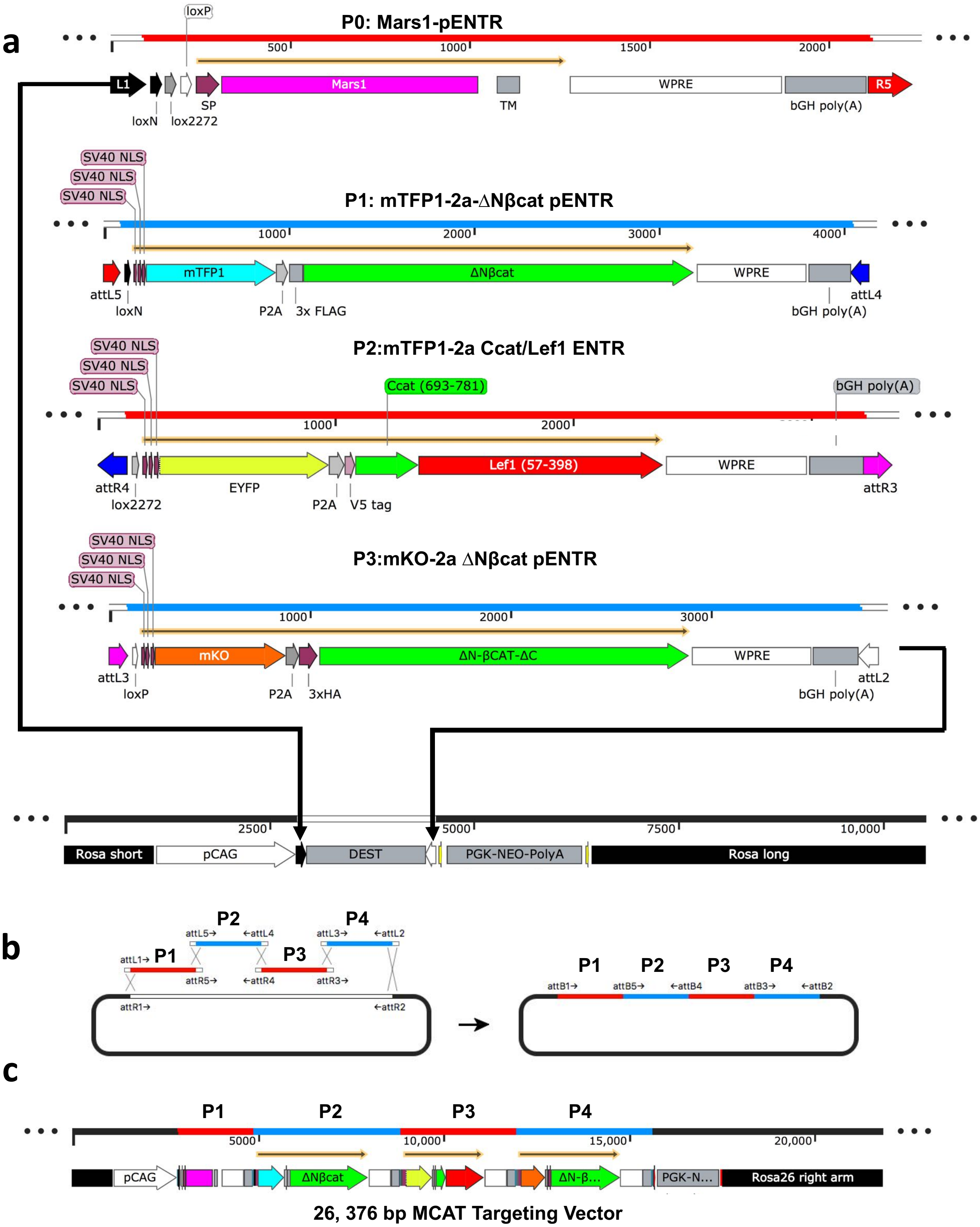

Supplementary Figure 5. Genetic map of MCAT Crainbow mice. (a) FTYK pENTR vectors were used to assemble a ROSA-targeting vector for ΔNßcat, Ccat/Lef1, and ΔNßCatΔC (MCAT). cDNA encoding 3x-FLAG-tagged and N-terminally truncated mouse ßcatenin (Uniprot Q02248, aa79-781) was Infusion® cloned into P2 pENTR (ΔNßcat). V5-tagged mouse ßcat C-terminal transactivation domain (Q02248, aa693-781) fused to N-terminally truncated mouse Lef1 (Uniprot P27782, aa57-398) was Infusion® cloned into P3 pENTR (Ccat/ Lef1). 3x-HA-tagged N-terminally and C-terminally truncated mouse ßcat (Uniprot Q02248, aa79-693) was Infusion® cloned into P4 pENTR (ΔNßCatΔC). **(b)** Single-step multi-fragment Gateway™ assembly of FTYK pENTR vectors from **"a"** into a previously described Gateway™-compatible ROSA-targeting mouse vector[1]. **(c)** ROSA MCAT Crainbow vectors were restriction mapped, sequence verified using next-generation sequencing (NGS), and then linearized for ES cell electroporation and selection.

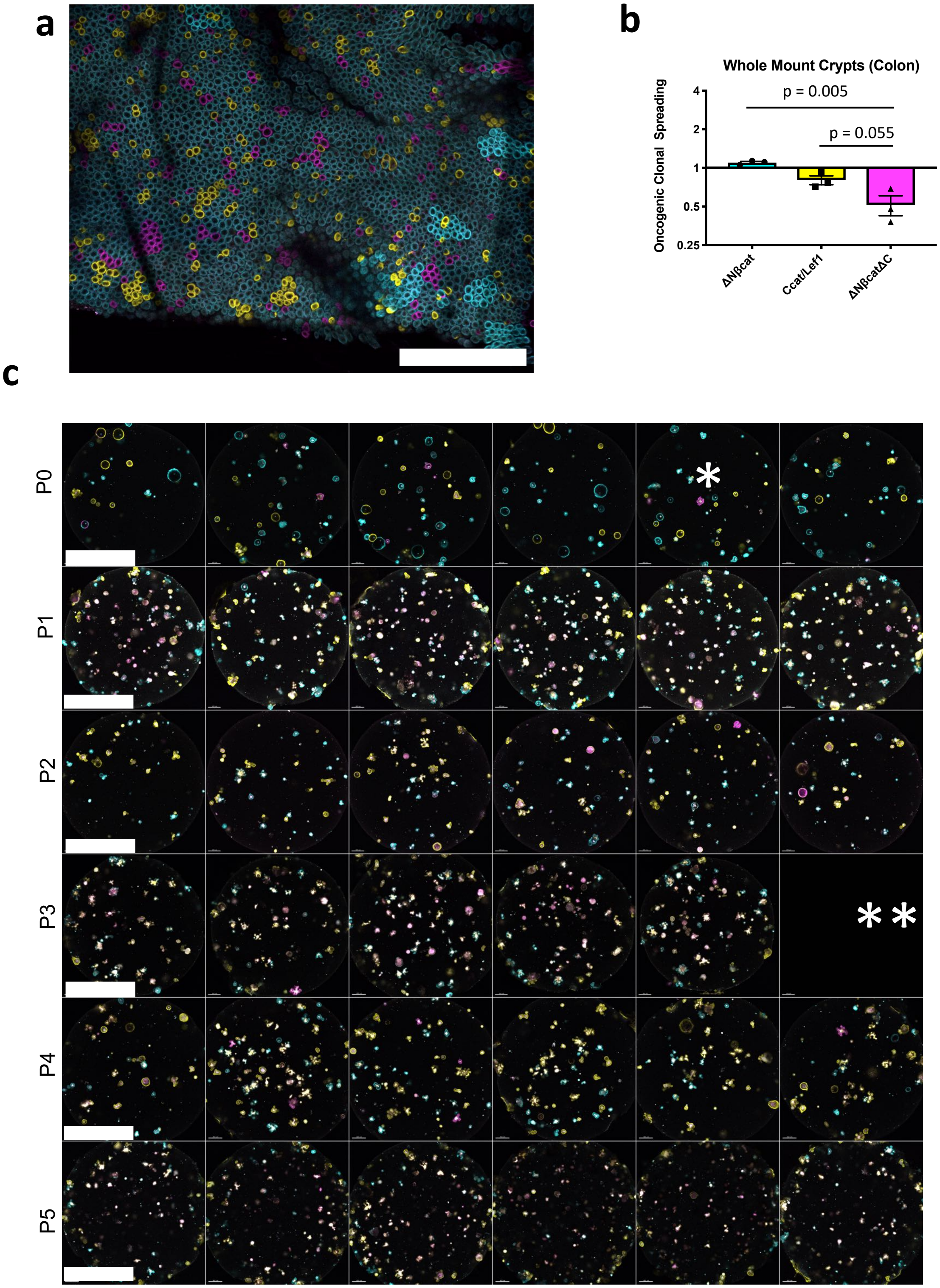

Supplementary Figure 6. Oncogenic Clonal Expansion in MCAT mice. (a) Wholemount confocal imaging of colon from $MCAT^{VilCre}$ mice (N=3 mice, 6 weeks of age, representative image shown). **(b)** Calculation of field cancerization potential in colon, as described in text and in Figure 3. p-values were calculated by one-way ANOVA and Tukey's post hoc testing. **(c)** Organoids were made from MCAT mice and imaged before passaging (N = 6 wells imaged, **N = 5 wells imaged at passage 3). P0 = parental organoids that were then passaged and imaged five times (P1-P5). Organoids were counted by color and normalized to the P0 count to quantify field spreading *ex vivo*. *Higher magnification of P0 was used in **Figure 3d.** Scale bars = 1mm in **a** and 2mm in **c**.

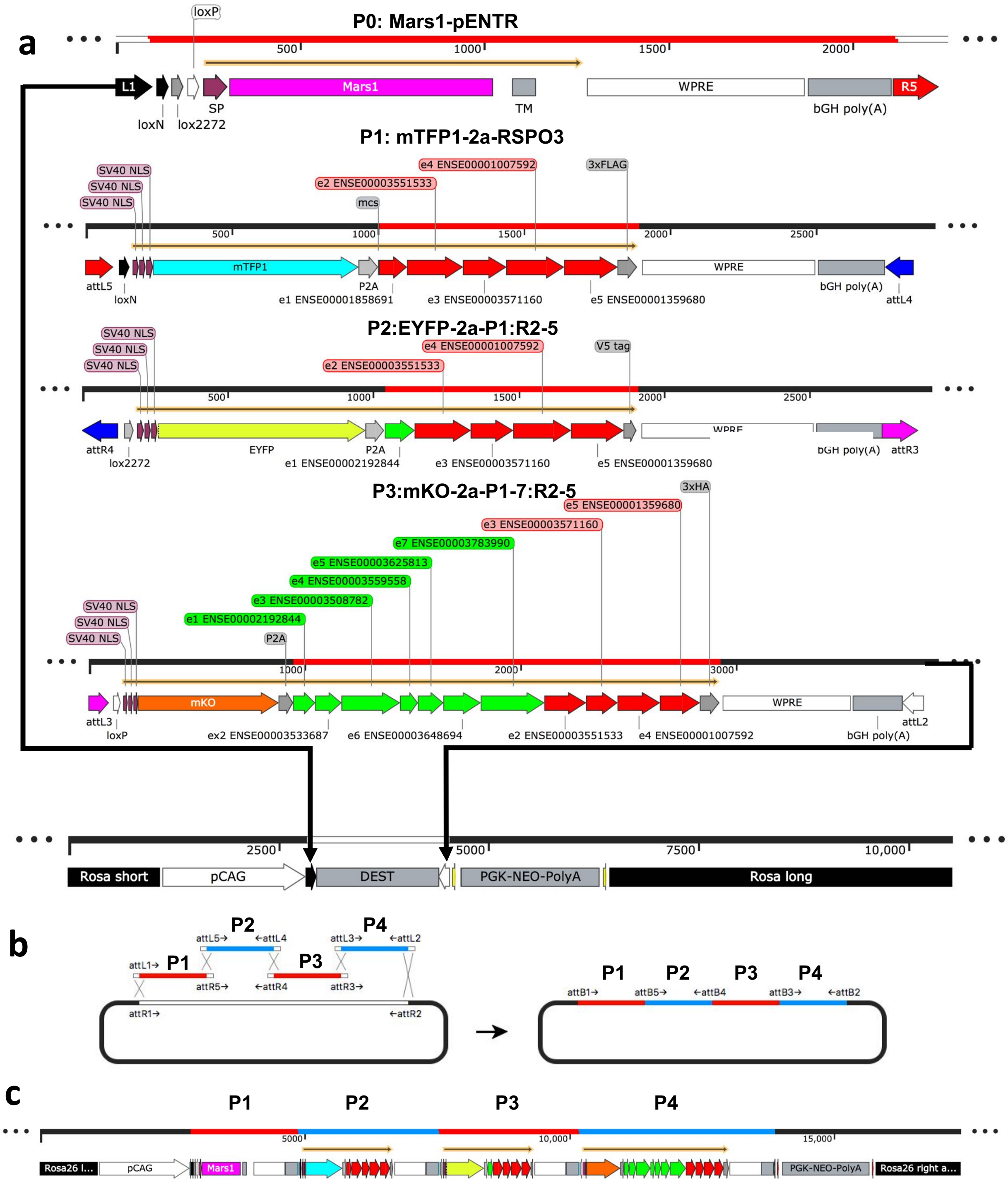

24, 636 bp ROBO Targeting Vector

Supplementary Figure 7. Genetic map of ROBO Crainbow mice. (a) FTYK pENTR vectors were used to assemble a ROSA-targeting vector for WT RSPO3 and PTPRK:RSPO3 fusions (ROBO). cDNA encoding 3x-FLAG-tagged WT human RSPO3 (Uniprot Q9BXY4, aa1-272) was Infusion® cloned into P2 pENTR (RSPO3). cDNA encoding V5-tagged human PTPRK^{e1}:RSPO3$^{e2\text{-}5}$ (Exon1 PTPRK: Uniprot Q15262, aa1-33 and Exons2-5 RSPO3: Uniprot Q9BXY4, aa34-272) was Infusion® cloned into P3 pENTR (P1:R2-5). cDNA encoding 3xHA-tagged human PTPRK$^{e1\text{-}7}$:RSPO3$^{e2\text{-}5}$ (Exon1-7 PTPRK: Uniprot Q15262, aa1-387 and Exons2-5 RSPO3: Uniprot Q9BXY4, aa34-272) was Infusion® cloned into P4 pENTR (P1-7:R2-5). **(b)** Single-step multi-fragment Gateway™ assembly of FTYK pENTR vectors from **"a"** into a previously described Gateway™-compatible ROSA-targeting mouse vector[1]. **(c)** ROSA ROBO Crainbow vectors were restriction mapped, sequence verified using next-generation sequencing (NGS), and then linearized for ES cell electroporation and selection.

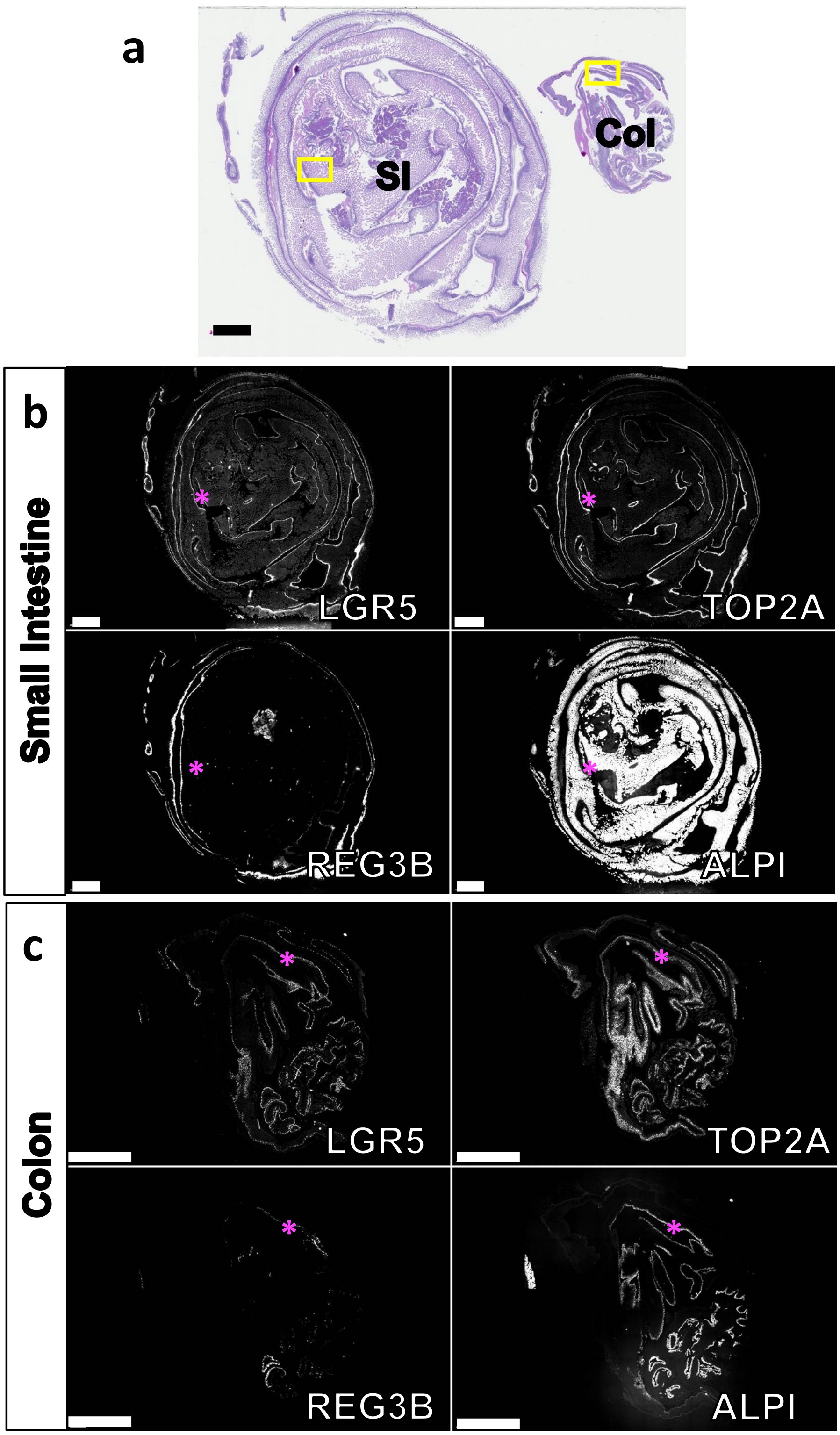

Supplementary Figure 8. RNA FISH and histopathology coregistry of WT small intestine and colon. (a) Swiss-rolled small intestines and colons from WT mice (PN18) were formalin fixed paraffin embedded (FFPE), sectioned, H&E stained, and whole-slide imaged (WSI). Multiprobe RNA FISH for *Lgr5/Top2a* and *Lgr5/Reg3b/Alpi* were performed on serial adjacent sections. Sections were WSI by confocal microscopy and are presented as greyscale for **(b)** small intestine and the **(c)** colon. The magenta asterisk denotes additional regions of interest evaluated at higher power in **Supplementary Figures 9-10 and Figure 5.** Scale bars = 2 mm.

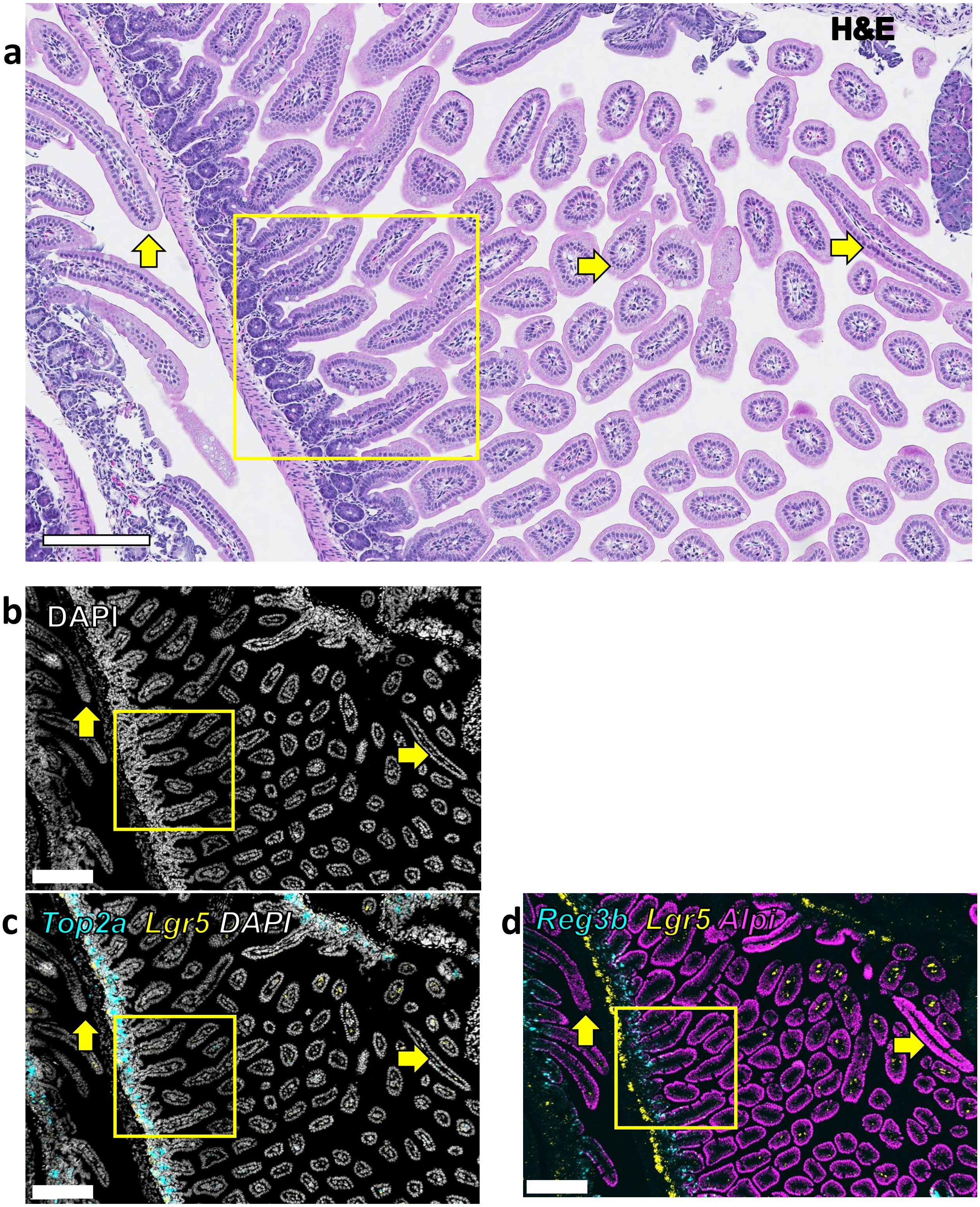

Supplementary Figure 9. Coregistry of WT small intestine. Higher magnification coregistry of the area in **Supplementary Figure 8** denoted by the magenta asterisk. The yellow arrowheads indicate landmarks for the coregistry of **(a)** H&E histopathology with **(b)** DAPI, **(c)** *Top2a* (cyan), and *Lgr5* (magenta), and **(d)** *Reg3b* (cyan), *Lgr5* (yellow), and *Alpi* (magenta). The regions of interest are presented at a higher power in **Figure 5** . Scale bars = 200μm.

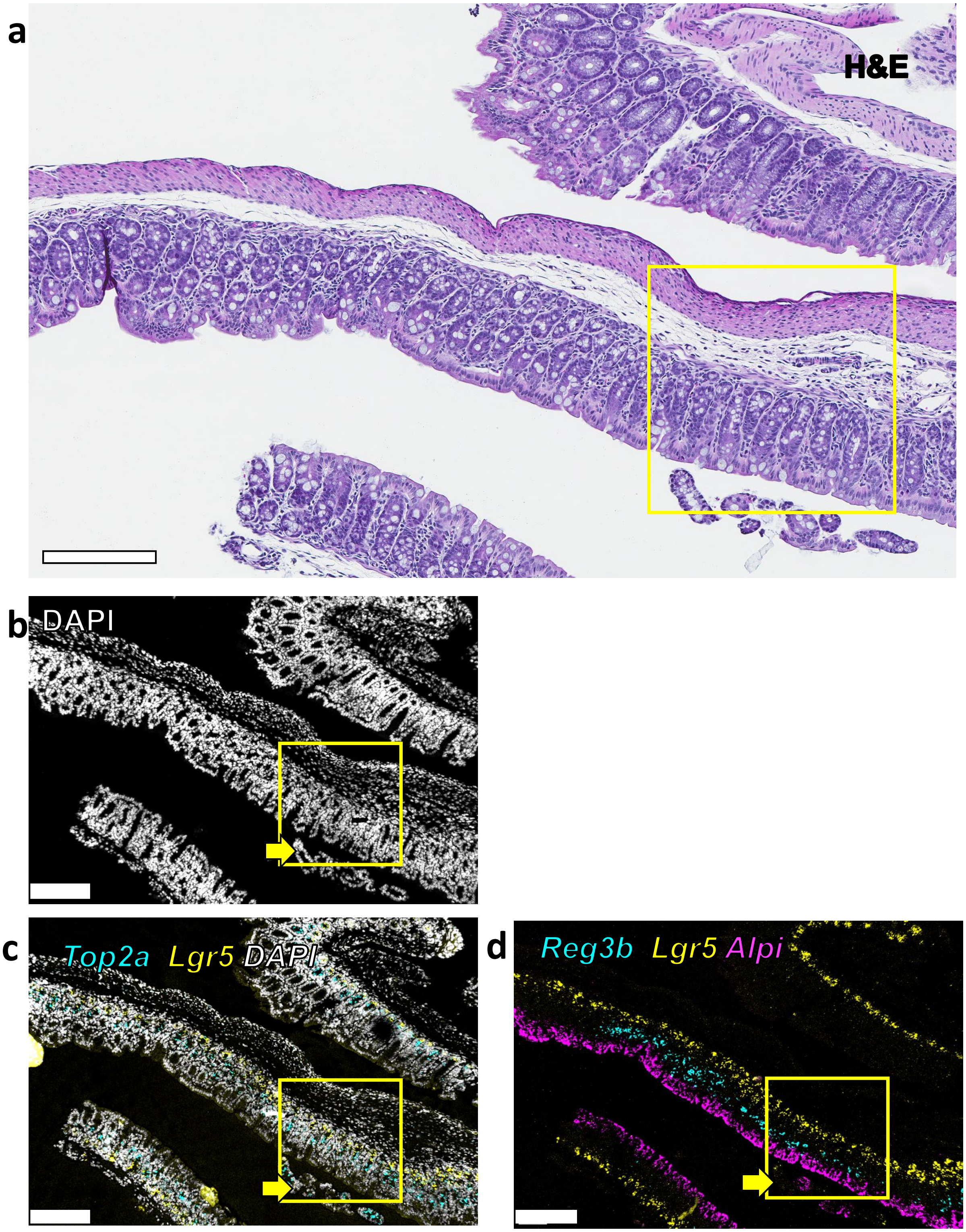

Supplementary Figure 10. Coregistry of WT colon.
Higher magnification coregistry of the area in **Supplementary Figure 8** denoted by the magenta asterisk. The yellow arrowheads indicate landmarks for the coregistry of **(a)** H&E histopathology with **(b)** DAPI, **(c)** *Top2a* (cyan), and *Lgr5* (magenta), and **(d)** *Reg3b* (cyan), *Lgr5* (yellow), and *Alpi* (magenta). The regions of interest are presented at a higher power in **Figure 5.** Scale bars = 200µm.

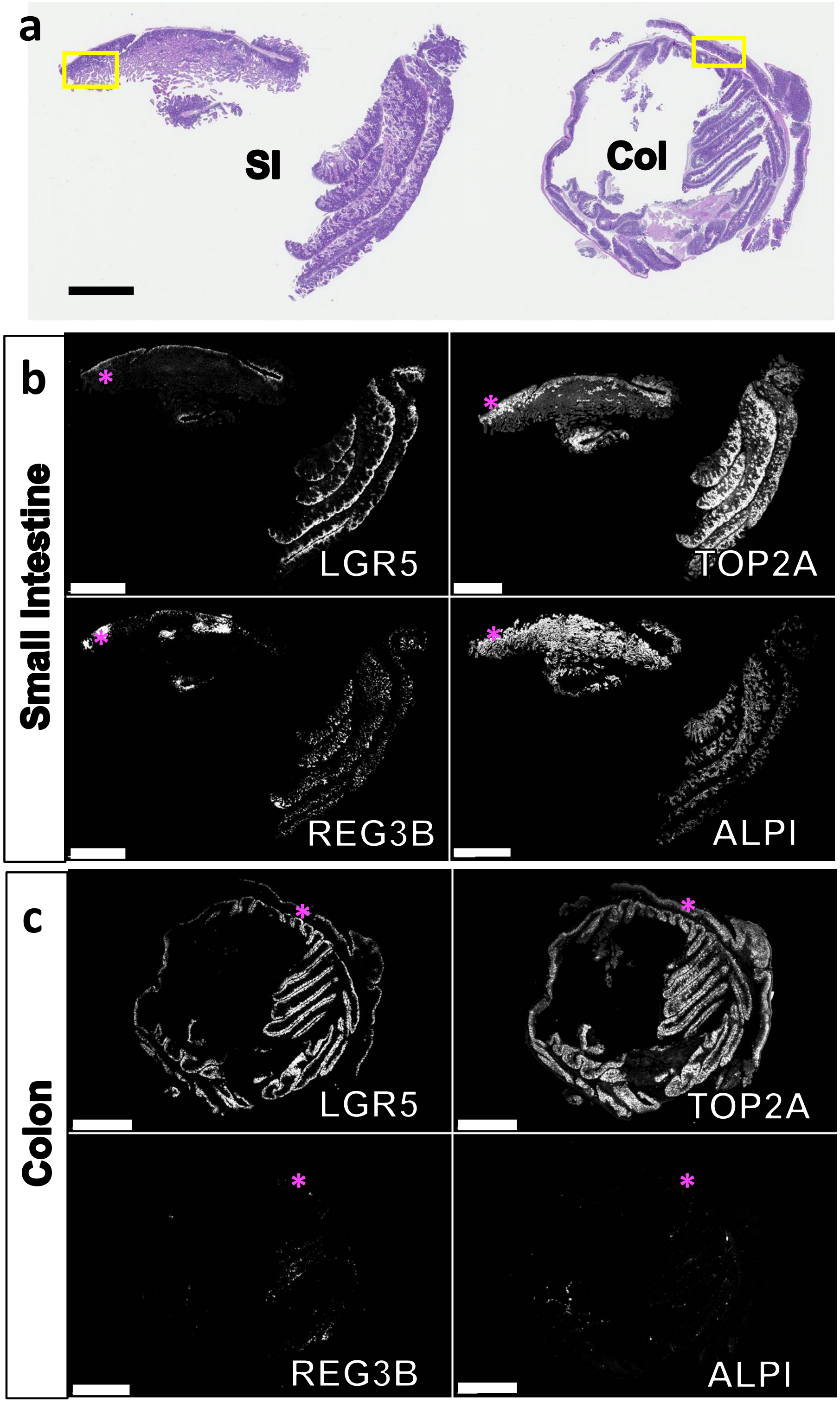

Supplementary Figure 11. RNA FISH and histopathology coregistry of ROBO small intestine and colon. **a)** Swiss-rolled small intestines and colons from ROBO mice (PN18) were formalin fixed paraffin embedded (FFPE), sectioned, H&E stained, and whole-slide imaged (WSI). Multiprobe RNA FISH for *Lgr5/Top2a* and *Lgr5/Reg3b/Alpi* were performed on serial adjacent sections. Sections were WSI by confocal microscopy and are presented as greyscale for the **(b)** small intestine and the **(c)** colon. The magenta asterisk denotes additional regions of interest evaluated at a higher power in **Supplementary Figures 12-13 and Figure 5.** Scale bars = 2 mm.

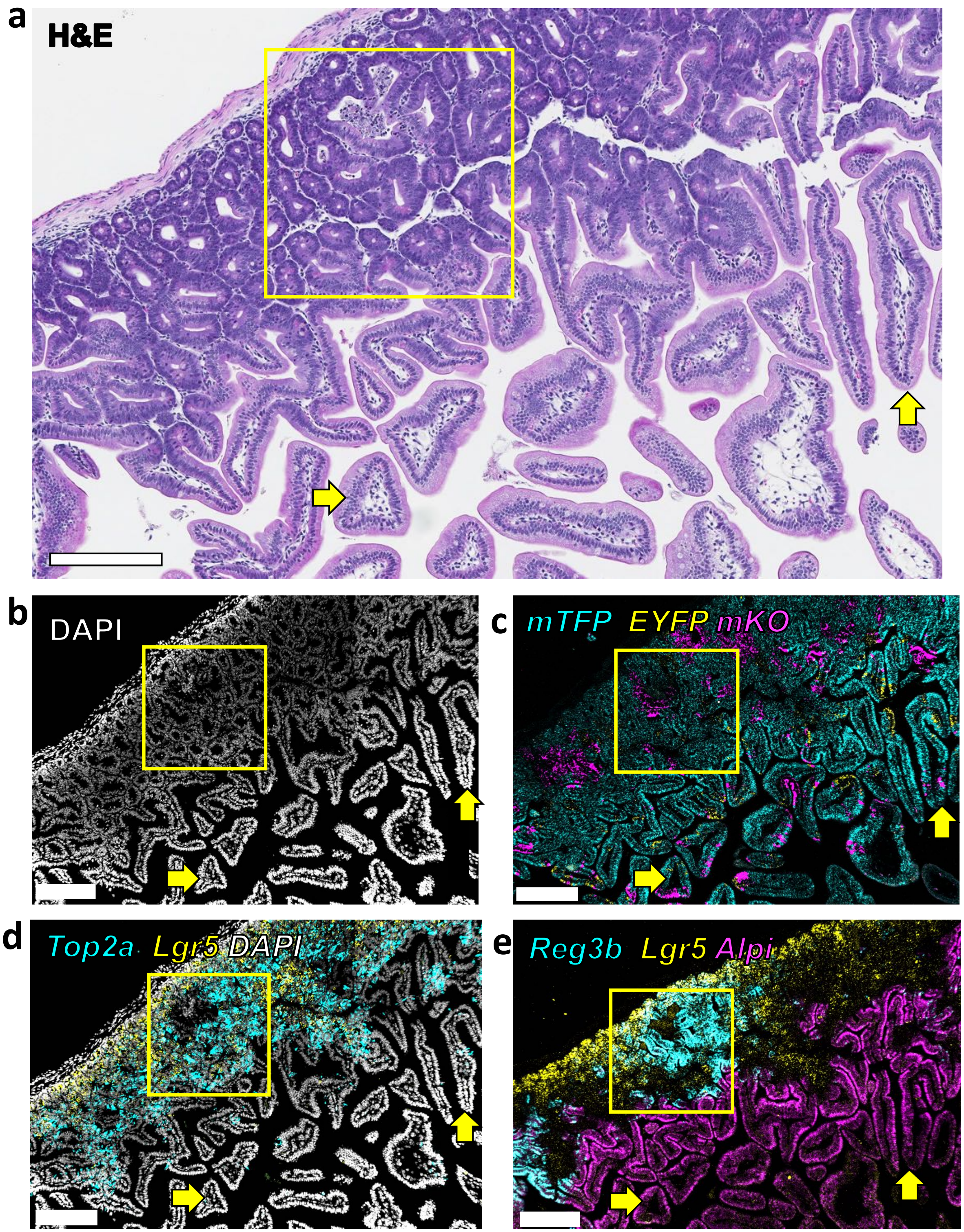

Supplementary Figure 12. Coregistry of ROBO small intestine. Higher magnification coregistry of the area in **Supplementary Figure 8** denoted by the magenta asterisk. The yellow arrowheads indicate landmarks for the coregistry of **(a)** H&E histopathology with **(b)** DAPI and multiprobe RNA FISH for **(c)** *mTFP1, EYFP,* and *mKO*, **(d);** Top2a (cyan) and *Lgr5* (magenta); and **(e)** *Reg3b* (cyan), *Lgr5* (yellow), and *Alpi* (magenta). The regions of interest are presented at a higher power in **Figure 5.** Scale bars = 200µm.

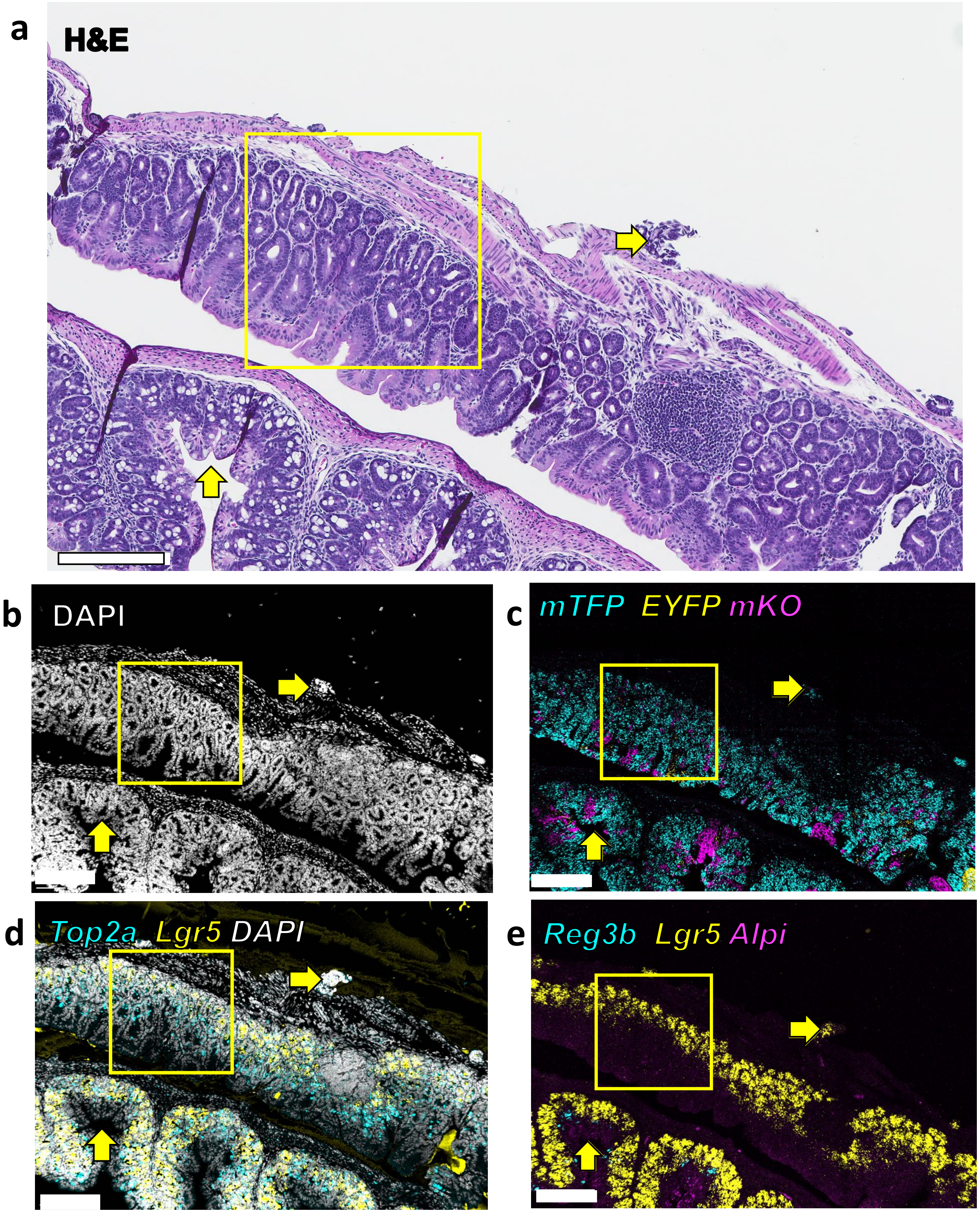

Supplementary Figure 13. Coregistry of ROBO colon. Higher magnification coregistry of the area in **Supplementary Figure 8** denoted by the magenta asterisk. The yellow arrowheads indicate landmarks for the coregistry of **(a)** H&E histopathology with **(b)** DAPI and multiprobe RNA FISH for **(c)** *mTFP1, EYFP,* and *mKO*; **(d)** Top2a (cyan) and *Lgr5* (magenta); and **(e)** *Reg3b* (cyan), *Lgr5* (yellow), and *Alpi* (magenta). The regions of interest are presented at a higher power in **Figure 5.** Scale bars = 200μm.

a

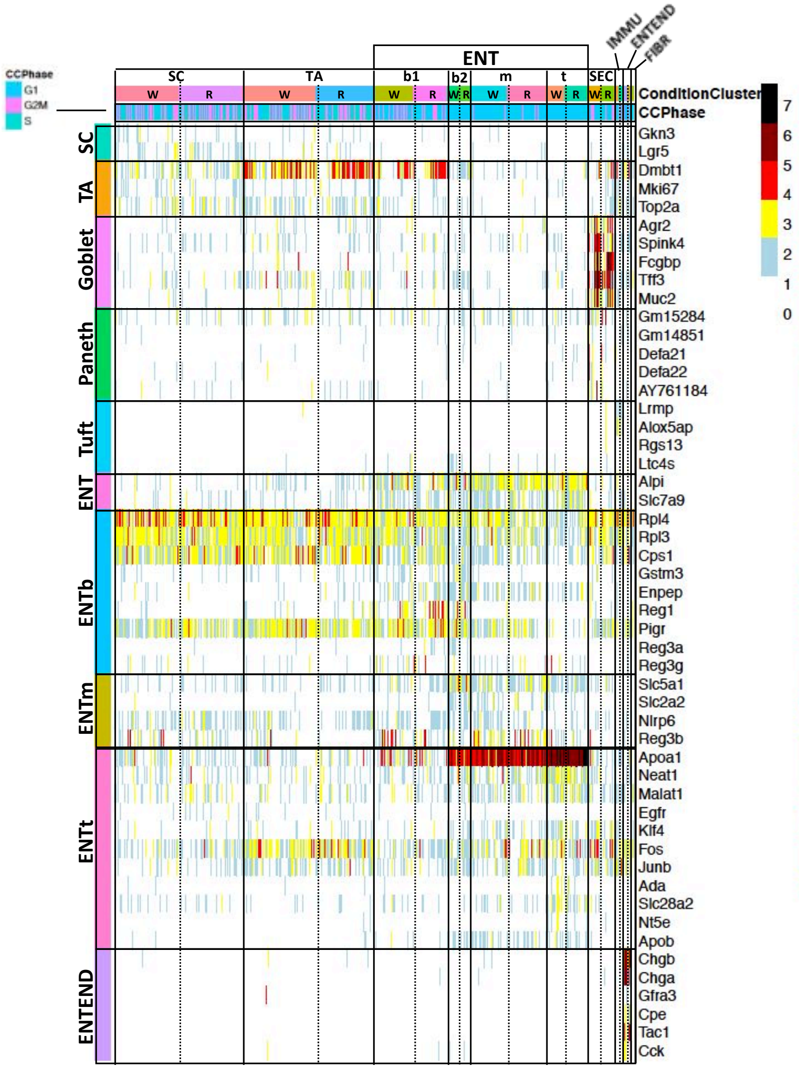

b

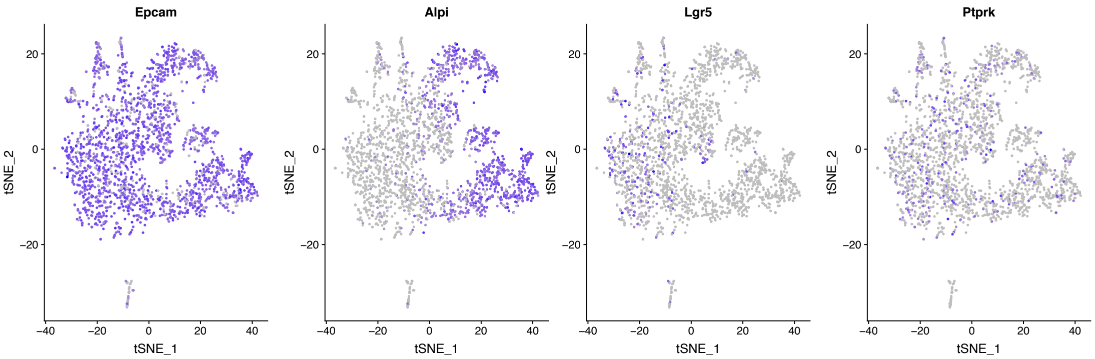

Supplementary Figure 14. scRNAseq analysis of WT and ROBO mouse intestinal crypt cells.
(a) Heat-map analysis of top marker genes and genes previouslyidentified by Moor, *et.al.*[2]. The top of the heatmap is annotated for cell-type and genotype (W:WT, and R:ROBO). Cell Cycle phase is displayed for each cell. Genes at the right are used as cell-type specific markers
(cell types annotated on the left).SC: Stem cell, TA: transiently amplifying cell, ENT:enterocytes that included bottom (b1: S/G2M phase and b2: G1), middle (m) and top (t) and were recently described [2], SECR: secretory Paneth and Goblet cells, IMMU: immune, ENTEND: enteroendocrine, FIBR (Fibroblast). **(b)** Feature plots of the epithelial marker “Epcam”, Enterocyte marker “Alpi”, Stem cell marker “Lgr5” and endogenous “Ptprk”. Ptprk is modestly expressed at throughout the epithelium.

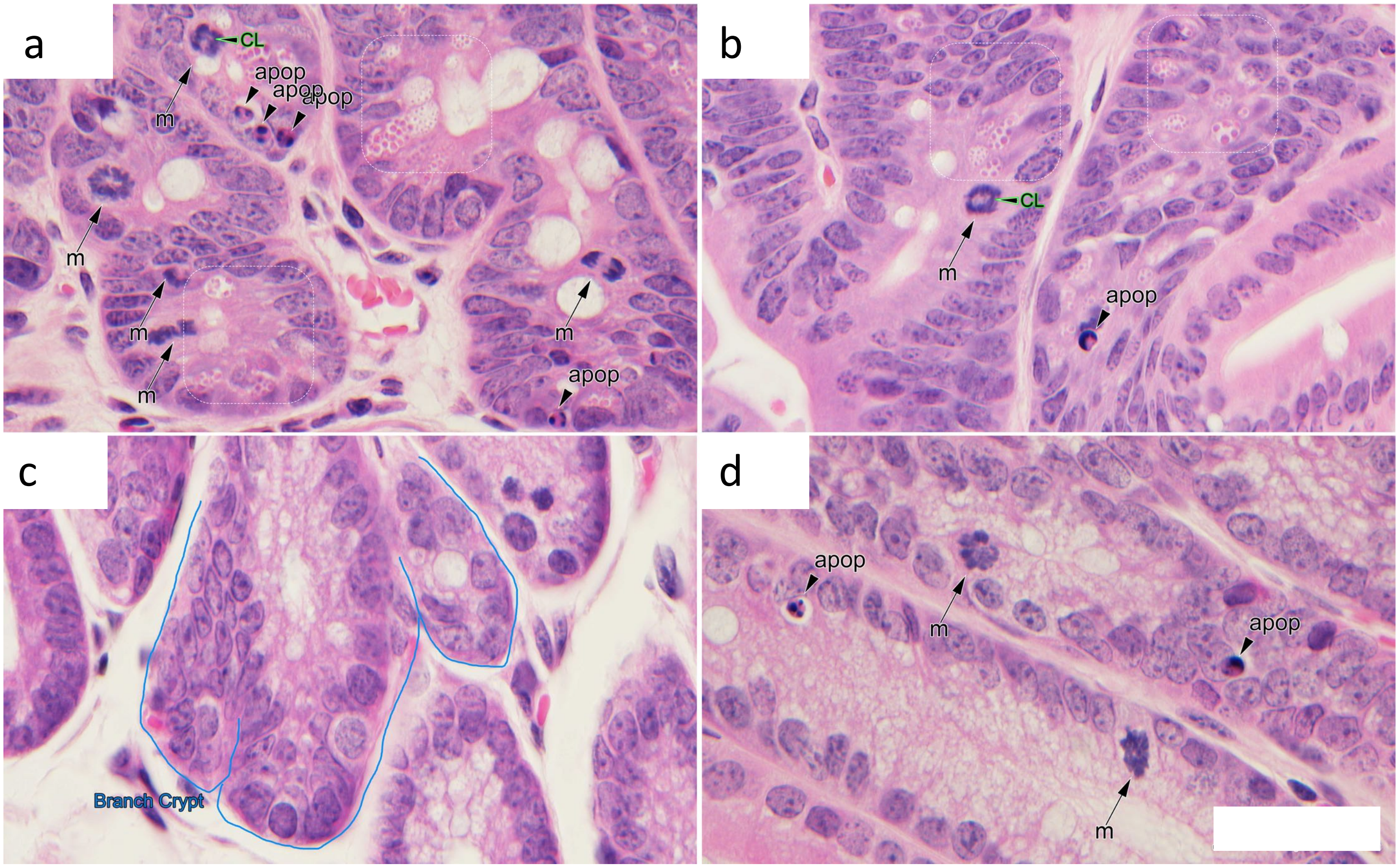

Supplementary Figure 15. Histopathology in adult ROBO$^{CreER/T2}$ Crainbow mice. (a-b) The small intestine has a diffuse field of crypt hyperplasia with increased undifferentiated crypt cells and increased Paneth-like cells (dashed boxes) with increased mitotic activity (m-tagged arrows). Crypts were irregular with branching and budding. Although maturation of the villi with typical luminal villous epithelial cells are observed with basally located nuclei, eosinophilic cytoplasm, and surface microvilli (brush-border), foci of the more undifferentiated crypt cells migrated up the villi. Many of these areas are dysplastic with increased mitoses with abnormal mitoses including “starburst” forms and chromosome lag (CL-tagged green arrowheads) and apoptotic figures (apop-tagged arrowheads), consistent with preneoplastic potential. **(c-d)** The large intestine was characterized by colonic crypt hyperplasia with increased undifferentiated luminal cells with corresponding decrease in mucin producing goblet cells, increased mitotic activity (m-tagged arrows), apoptosis (apop-tagged arrowheads) and crypt architectural distortion (panel C; blue outline labeled “Branch Crypt”). Scale bar = 50μm.

a

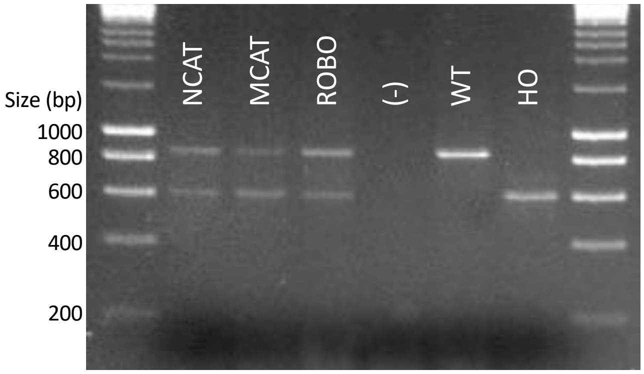

RTg_F	AGCACGTTTCCGACTTGAGTTG
RTg_R	CACTTGATGTACTGCCAAGTGG
WT_R	GAAAATGCCAATGCTCTGTCTAGG

b

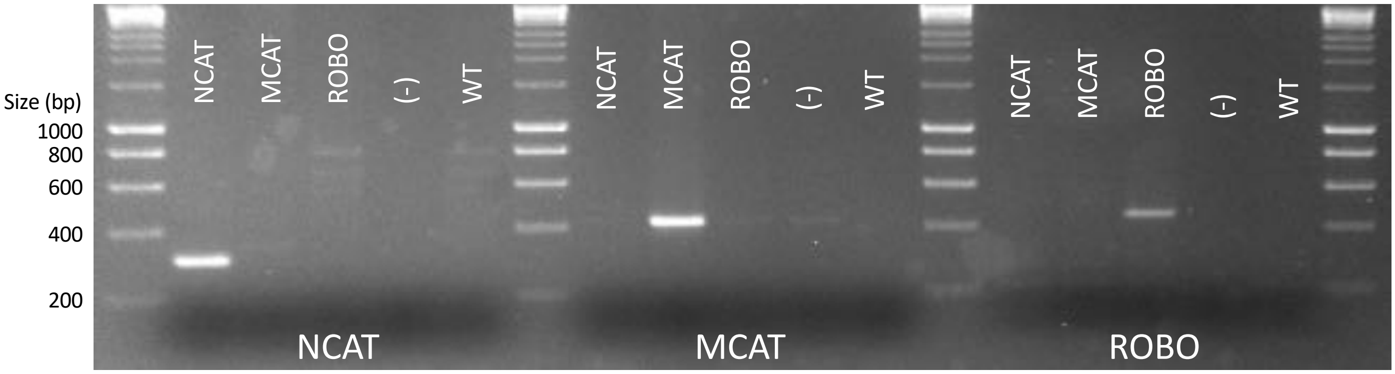

NCAT_F	AGAAGTGCACGCGTGAGTAAGC
NCAT_R	CTGGACATTAGTGGGATGAGC
MCAT_F	GCATGGACGAGCTGTACAAG
MCAT _R	TTGCTGGCTGGGATGATTTCG
ROBO_F	TTAACTTTCCTGCCGATGGTCC
ROBO_R	CATCAAAAGTACAGCCACCTGC

c

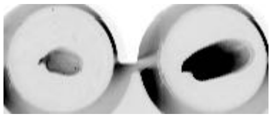

WT Tg +

Supplementary Figure 16. Crainbow genotyping. (a) PCR genotyping results for the universal detection of Crainbow transgenes in the mouse ROSA locus. The indicated primer set produces an 837-bp band for WT ROSA locus and a 600-bp band for the Crainbow transgene. **(b)** Crainbow-specific genotyping for NCAT, MCAT, and ROBO mice using the indicated primer sets. **(c)** SCi1 staining for FAP-Mars1 (position 0 of transgene) in toes from WT or Tg+ mice. Fresh toe biopsies were incubated with SCi1 (1:2000) in 0.2% Triton X-100 overnight, shaking, at 4°C. Toes were imaged on a Li-Cor Odyssey Classic with a 700-nm excitation wavelength.

Supplementary References

1 Snyder, J. C. *et al.* Inhibiting clathrin-mediated endocytosis of the leucine-rich G protein-coupled Receptor-5 diminishes cell fitness. *J Biol Chem*, doi:10.1074/jbc.M116.756635 (2017).

2 Moor, A. E. *et al.* Spatial Reconstruction of Single Enterocytes Uncovers Broad Zonation along the Intestinal Villus Axis. *Cell* **175**, 1156-1167 e1115, doi:10.1016/j.cell.2018.08.063 (2018).
