# Supplementary material for: A cancer rainbow mouse for visualizing the functional genomics of oncogenic clonal expansion
Source: Nat Commun. 2019 Dec 2;10:5490. doi: 10.1038/s41467-019-13330-y (PMC6889384; doi:10.1038/s41467-019-13330-y)
Supplement: Supplementary file 3 — Description of Additional Supplementary Files [file 41467_2019_13330_MOESM3_ESM.pdf]

Description of Additional Supplementary Files

File Name: Supplementary Data 1
Description: NCAT ROSA-targeting plasmid. Annotated sequence provided in GenBank file format.

File Name: Supplementary Data 2
Description: MCAT ROSA-targeting plasmid. Annotated sequence provided in GenBank file format.

File Name: Supplementary Data 3
Description: ROBO ROSA-targeting plasmid. Annotated sequence provided in GenBank file format.

File Name: Supplementary Data 4
Description: scRNAseq DIGE analysis. Differentially expressed genes ($p<0.01$) were assessed between WT and ROBO cell-types.
